# Artificial intelligence and biological research

**DOI:** 10.1093/nsr/nwae415

**Published:** 2024-11-28

**Authors:** Chung-I Wu, Cai Li

**Affiliations:** School of Life Sciences, Sun Yat-sen University, China

While artificial intelligence (AI) is becoming ubiquitously influential in our society, its role in research is not that obvious. After all, research is about innovation, demanding that scientists defy their training when necessary. That is how new paradigms come to be. AI, on the other hand, depends greatly on the scale of training, which might (or might not) hinder innovation. How soon may AI evolve into innovators? One may thus wonder whether, or when, AI would come up with the theory of plate tectonics if it was fed all the data up to the late 1950s. This is intriguing, as the theory of continental drift was accepted only after the proposal of plate tectonics.

In biology, it is conceivable that AI ‘might’ be able to develop the theory of neutral molecular evolution. When Motoo Kimura formally proposed the theory in 1968 [1], it was originally based on the observations of too much evolution and too much protein diversity vis-à-vis the conventional calculations. AI might be able to follow the logic of mutation load and genetic death to figure out the contradiction. Although such arguments are no longer used, it was the amount of data that was available in the form of protein sequences that led to the revolutionary idea. This part of history is thus relevant in the era of AI and genomics.

With the torrent of genomic data, the six perspectives in this special topic provide an up-to-date glance at what can be done in the interface of genes/genomics and AI. Briefly, all these perspectives introduce the logic flow of [input data]—[AI model (e.g. neural network)]—[output responses]. For example, a biologist may ask: With the input of the transcriptomic data as well as a set of siRNAs, can we infer the resultant expression profile and, possibly, the cell fates? In this special topic, the authors focus on AI models. Nevertheless, they also provide examples of the inputs and outputs that concern biological research the most.

The crux of AI, of course, is the machinery between inputs and outputs. From these perspectives, biologists may realize that AI modeling is drastically different from the conceptual models that they are used to. For example, one may conceptualize the gene regulatory network from the May-Wigner theory [2], as it would tell us whether the network is stable or not. Similarly, to model molecular evolution, one may think of the diffusion model or the coalescence process that inform us about the evolutionary dynamics of mutations. The main difference is a conceptual approach vs. an empirical one. AI appears to be an empirical approach based on extensive training to connecting input and output. Usually, little or limited explicit modeling based on the domain-specific knowledge is incorporated into the learning machine. An earlier study indeed compares AI to generalized, non-linear regression of very high dimensions [3]. By and large, current AI can excel in predictive tasks that are often deficient in interpretability.

In this special topic, Bian *et al.* [4] and Yu and Zhang [5] review different foundation models to cover single-cell transcriptomics and transcription regulation, respectively. Bian *et al.*, based on a recent publication [6], present a good example of explicit descriptions of inputs and outputs. This foundation model uses 50 million single-cell transcriptomes as the input, transformed with 100 million parameters, and delivers outputs of drug responses and single-cell perturbation prediction, cell-type annotation and gene-module inference. Yu and Zhang discuss the role of large language models in modeling and understanding transcription regulation. It also explores the potential of developing multimodality foundation models to integrate diverse input data types for more accurate transcriptional predictions and further insights into regulatory mechanisms.

Li *et al.* [7] and Zhang *et al.* [8] cover the biomolecular structure and sequence design that are at the forefront of AI biology and play important roles in drug discovery and synthetic biology. Li *et al.* discusses the evolution and advancements of deep-learning methods in modeling biomolecular structures, particularly focusing on generalized models such as AlphaFold3 and RFAA in predicting complex structures involving proteins, nucleic acids and small molecules. Zhang *et al.* discuss how AI-guided strategies are transforming the paradigm of biological sequence design. By combining generative and predictive AI models, these methods can perform digital experiments that accelerate evolutionary processes to obtain desirable sequence candidates.

Wang and Ye [9] review recent advancements in deep-learning-based genomic variant callers, highlighting their superior accuracy in detecting small and large genomic variants. Notably, these models encode the input genomic data into images and transform variant calling problems into computer vision problems that have been extensively addressed in computer science. Xiao and Zhao [10] discuss how various AI methods, from traditional machine learning to deep learning and emerging frontiers such as large language models and multimodal integration, are improving the efficiency and accuracy of identifying microbiome-based biomarkers.

These six perspectives belong in the pioneering efforts that usher AI into biological research. It is very likely that the trend will continue at an accelerating rate.
